# Attitudes of elderly Austrians towards new technologies: communication and entertainment versus health and support use

**DOI:** 10.1007/s10433-019-00508-y

**Published:** 2019-04-05

**Authors:** Nicole Halmdienst, Michael Radhuber, Rudolf Winter-Ebmer

**Affiliations:** Johannes Kepler University, Linz, Austria

**Keywords:** Health support, Technology, Entertainment

## Abstract

We use data from SHARE (The Survey of Health, Ageing and Retirement in Europe) in Austria to investigate attitudes towards new technologies in information and communication technology. The technologies can significantly facilitate the daily lives of an ageing population. In Austria, in wave 6 in 2015, an additional paper-and-pencil questionnaire was implemented which asked details about attitudes towards different technological innovations. From these questions, we develop a binary attitude score which indicates positive attitudes towards new technologies. In probit estimations, the attitude score is related to different demographic and health variables. Our main results indicate that strong gender differences in attitudes towards new technologies exist: men value communication and entertainment devices more, whereas women’s attitudes are more positive towards devices that include a specific health or support value. Furthermore, while older cohorts value entertainment devices less than younger ones, no such pattern exists for health and support systems.

## Introduction

Information and communication technology (ICT) can potentially facilitate the daily lives of an ageing population. Several technological innovations were proposed and tested in prototypical situations in the domain of communication and entertainment, as well as in situations in which individuals require help in health-threatening situations. There is a paucity of systematic studies on the attitudes of elderly individuals above 50 years of age towards such technologies, and thus, we investigate the same by using a representative sample of individuals $50+$ living in Austria participating in the Survey on Health, Ageing and Retirement in Europe (SHARE).

In an environment with a growing old-age dependency ratio, not all individuals are able to request or afford personal assistance whenever required (Choi et al. 2014; Tennant et al. 2015). New technology potentially offers solutions in cases of still highly autonomous individuals or couples, who nevertheless require a certain amount of assistance or supervision in their daily living activities (Blackman et al. 2016). The objective of resorting to new technologies primarily corresponds to enabling these individuals to continue their daily activities and lives for as long as possible independently, while ensuring that they are offered the necessary technological assistance to cope with their (personal) needs. Technology potentially offers a solution for increasing the quality of life of elderly persons while relieving social systems from (some of) demographic pressure.

The existence of technological possibilities, such as robots working and serving in nursing homes or artificial intelligence providing automatic language translation between nurses and patients (to name just two recent examples), is not sufficient to initiate the broad implementation of such technologies (See e.g. Joe and Demiris 2013 for a review of feasibility studies of the use of mobile phones for health; most of the studies use samples involving 10–20 individuals.). Specifically, with respect to individuals in older age groups, technological scepticism prevails, and the usability of new technologies is an important issue (See e.g. Grindrod et al. 2018 for an example of usability problems in authentication options for mobile phones for older adults.).

One of the biggest obstacles to the introduction of the aforementioned types of technological innovations is undoubtedly the personal attitude of ageing individuals and their willingness to confront new technological devices (Ma et al. 2015). Our study examines differences in attitudes between communication and entertainment usage as well as in health and support use. It is expected that aspects related to increased age and health limitations as intervening factors will be important.

## Previous research

Fang et al. (2018) have carried out a detailed literature review, exploring the digital divide for information and communication technologies (ICTs) in general. Their review is based on the resources and appropriation theory by Van Dijk (2012) that encompasses mental, material, social, cultural, and temporal contexts, as well as the intersectionality framework by Hancock (2007). They stress the importance of the sociodemographic context, such as education, income, age, and gender, as a driver for nonuse and nonaccess to the internet. Accordingly, persons with advantaged positioning, such as the educated, white and upper class members without markers of inequity, are more likely to have access to the internet and use it more regularly than their counterparts, such as persons with limited or no education, non-whites and working class members, possibly with other markers of inequity. Significant effects regarding ICT use are therefore expected for variables such as education, income, and age; generational effects such as having children and a partner possibly are important confounders for aforementioned variables, while the evidence presented for gender is rather mixed, however.

Other authors have focused on the use of communication technology in older age. Gell et al. (2013) analysed data from the U.S. 2011 National Health and Ageing Trends Study (NHATS) with approximately 8000 observations $65+$. The main purpose was to determine patterns fostering the use of communication technology such as email, internet, and text messaging. Based on their results, the use of technology is related to age, gender, race, educational level, and marriage status. Young age, male gender, white race, higher education, and being married are correlated with increased use of technology. The use of technology decreases significantly with physical and mental limitations. In a similar way, Vorrink et al. (2017) established that technology use (for e.g. broad variety of 33 technological items: computer, fax, smartphone, mobile phone, tablet, email, navigation system, video phone, e-reader, and fitness device) is negatively associated with age, lower education, lower social status (income), and lower physical functioning. With respect to gender and employment status, significant effects were not observed. Their sample is based on Dutch respondents over the age of 65 years. Ma et al. (2016) investigated personal factors affecting the acceptance of smartphone technology by older Chinese adults. Unfortunately, their sample was constrained to individuals below the age of 65 years.

Further research is dealing with associations between frailty and use of technology. With Finnish data, the use of information and communication technologies (ICT) in the context of frailty, which is an indicator for health and independence, is being focused by Keränen et al. (2017). They examined disparities in internet usage between individuals with different frailty levels. Their results indicate that frail individuals are less likely to possess access to internet (80% non-frail, 70% pre-frail, and 46% frail individuals enjoy internet connections at home). Frail individuals are also less likely to use tablets or smartphones. The individuals that continue to use such technology experience more difficulties when compared to non-frail individuals. Age is significantly negatively related with internet usage and use of tablets and smartphones. Higher education positively affects the use of information and communication technologies. Significant gender differences were not observed.

Other studies looked, in particular, at technological usability in relation to age. Mostaghel and Oghazi (2017) highlighted the importance of usefulness and ease of use of new technologies in terms of the acceptance by elderly individuals, with a sample of 800 individuals aged 60+ in Sweden. They observed that the ease of use of technological devices is significantly related to—mainly—age-related factors; although unease towards the usage of new technologies, cognitive ability and the ability to follow instructions appear as important drivers behind those age-related factors. A comfortable live and cognitive ability increase the perceived ease of use and usefulness of a technology, as does the ability to follow instructions. Czaja and Lee (2007) conversely indicated that older individuals are generally ready to use the advantages of new technologies. However, this is not observed in reality and is mainly due to usability issues and the availability of support. Cognitive functioning may play a crucial role here.

Using a similar data set as ours, Chopik (2016) used data from the Health and Retirement Study (HRS) in the United States in 2012 to investigate technology use for social connectedness, such as email, social networks, video or phone calls or smartphones, and health relations. In the HRS, older adults generally exhibit a positive attitude towards new social technologies. The study indicated that social technology use is associated with better health and well-being. Loneliness was lined out as a mediating factor, and it was argued that the elderly benefits from technology use because it decreases loneliness by simplifying communication. The findings are in line with those of another study on a smaller sample: Morton et al. (2018) investigated whether Internet connectivity and training in its use for social purposes support the well-being of older adults receiving care. The results indicate that Internet access and training support the self and social connectedness of vulnerable older adults and contribute positively to well-being. However, these studies did not look at communication and health-enhancing technology contemporaneously, as do we.

While technical capabilities are mostly reviewed in technical studies, actual use of such technologies in the older generation is largely dependent on acceptance, adaptability, usability, and assistance by younger relatives. There is a paucity of specific or large-scale quantitative analysis on the attitude towards or acceptance of technological security devices. However, numerous qualitative and small application studies indicate that the acceptance of the systems is considerably high (see e.g. Feldwieser et al. 2016; Claes et al. 2015). Tracking systems are often used as a backup to determine an individual with dementia in the case of wandering. The system is specifically important to care givers as a backup. White et al. (2010) indicated that it is often not elderly individuals themselves who decide to use the advantage of a GPS-tracking system (see also Landau et al. 2009).

From the existing literature, we would therefore expect characteristics as age, gender, education, income, physical and mental health (including cognitive abilities) to predetermine acceptability and use of new technologies, hence to be the primary catalysts for technology acceptance and use. Social status, housing and employment characteristics, race, marriage, and (grand-)children would possibly act as motivators for new technologies, facilitating or hampering access to and acceptance of technological innovations.

## Data and sample

Data for the study stem primarily from the SHARE Wave 6 survey in Austria, data release 6.0.0. The Survey of Health, Ageing and Retirement in Europe (SHARE) is a multidisciplinary and cross-national panel database of microdata on health, socio-economic status, and social and family networks of more than 120,000 individuals aged 50 years or older (more than 297,000 interviews). SHARE currently covers 27 European countries and Israel. The 6th wave of SHARE was implemented from January to September 2015. In Austria, 3.402 individuals were re-interviewed, and for 159 deceased respondents, an end-of-life interview was conducted in that longitudinal wave resulting in an individual response rate of 82% (Börsch-Supan and Malter 2017). Additionally, a country-specific paper-and-pencil questionnaire was implemented and covered several questions including two questions that focus on respondents’ attitudes towards and use of new technologies. The additional paper-and-pencil questionnaire was handed out to every regular SHARE respondent in Austria after the SHARE interview.

The paper-and-pencil questionnaire was developed by the authors at the Johannes Kepler University of Linz in cooperation with one of the funders of the SHARE survey in Austria, the Federal Ministry of Labor, Social Affairs, Health and Consumer Protection. A part of the paper-and-pencil questionnaire focusses on questions with respect to technology usage of an ageing population.^1^ It was designed and implemented exclusively in Austria. In total, 3.103 respondents aged 50 years or older returned the national paper questionnaire after the main interview was completed, and this resulted in a response rate of 91%.

SHARE is a panel survey. Sampling errors, non-response, and panel attrition therefore bias the representativity of the panel. Particularly in Austria, where the last refreshment sample was drawn in wave 4 in 2011, the youngest cohorts are underrepresented. In order to avoid the aforementioned problems, data were weighted with calibrated individual probability weights from the 6th wave of SHARE (Börsch-Supan and Malter 2017). Probability weights are available for a total of 3085 respondents. This results in a final sample size of 3085 that is used for analysis as cross-sectional data. According to the principles laid out by Solon et al. (2015), analysis beyond descriptive statistics is generally implemented both in a weighted and unweighted manner to control the model-misspecification and possible heteroskedasticity of the independent variables due to unobserved group-level factors. In the sample, 59% of respondents used for analysis are women. The average age is 69 years; 16% of respondents are younger than 60, 36% are between 60 and 69 years, and 33% belong to the age group 70–79.

## Methods

### Attitude score

The main data for the study originate from questions of the paper-and-pencil questionnaire from the SHARE wave 6 survey in Austria. We asked respondents if they were aware about a few new technological devices or innovations and asked questions on the respondents’ attitudes towards the same. With respect to the question on attitudes towards new technologies, respondents were asked to rate 11 different new technologies by selecting eight given different statements. The different technologies are as follows: Tablets, Smartphones, Social Networks, Voice-Controlled PCs, Emergency Tracking Systems,^2^ Auto Fall Alert devices, Personal Alarms, and Auto Cooker Control systems. Each new technology was evaluated with the following statements: ‘I do not know this’, ‘I am already using this’, ‘I am open to this’, ‘This is/would be a great help for me’, ’I find this daunting’, ‘I doubt that I would find this helpful’, ‘I am not interested in this’, and ‘I do not feel comfortable around this’ (see Table 3 in the Appendix). Multiple answers were possible.

From the question that focuses on respondents’ attitudes towards new technology devices, we built an attitude score in the form of a dichotomous variable that assumes the value of one in case of a positive statement and 0 for negative statements. With respect to positive statements on the attitude towards new technologies, we count ‘I am already using this’, ‘I am open to this’, and ‘This is/would be a great help for me’. Negative statements include ‘I find this daunting’, ‘I doubt that I would find this helpful’, ‘I am not interested in this’, and ‘I do not feel comfortable around this’. The statement ‘I do not know this’ is kept neutral, and thus is set to a missing value and excluded from our analysis. Furthermore, with respect to the cases in which respondents selected multiple responses, in our definition contradictory statements for the same item, the attitude score was set as missing and such occurrences were excluded from our successive analysis.^3^

#### Grouped attitude score

Based on first results on age trends presented in the following Sect. 5 and for the purposes of simplicity, we grouped all devices into the following two categories: *Communication and entertainment* and *support and health* devices. *Tablet*, *Smartphone*, *Social Networks,* and *Voice-Controlled PC* fall in the category *communication and entertainment*, and *Tracking System*, *Auto Fall Alert*, *Personal Alarm,* and *Auto Cooker Control* are categorized as devices in the *support and health* group. For the two groups of devices, we define a binary variable that assumes the value of one whenever at least one of the devices in that group is rated positively.

### Independent variables

The data were successively enriched with demographic and other information collected during the standard SHARE interview. Our main focus was on demographic variables, such as age, gender, education, employment status (not employed/white collar/blue collar), living circumstances (financial distress/living in urban/rural area, in a house/flat), and family context (living with a partner, having children), and also health-related variables such as self-rated health and instrumental limitations in activities of daily living (IADL).

The variable age measures the age of the respondent in years in the year of the interview. Gender is a binary variable, taking the value 1 in case of female gender. The variables higher education, not employed, white-collar worker, blue-collar worker, living in urban area, living in a house, partner lives in household, and has children are all dichotomous, taking the value one in case of ’applies’. The variable financial distress serves as proxy to additional control for the income situation of the household where the respondent lives. Finanical distress is derived from the question “Thinking of your household’s total monthly income, would you say that your household is able to make ends meet...(1) with great difficulty, (2) with some difficulty, (3) fairly easy, or (4) easily?” Since in SHARE, this question is only asked to the financial respondent of a household, we use the imputed form to have the information for every observation in the sample. For analysis, it is transformed into a binary variable taking the value one in case of only being able to make ends meet in case of some or great difficulty.

The originally ordinal, self-rated health variable is recoded into a binary variable taking the value 1 when fair or poor health was selected. The variable IADL (Instrumental Activitites of Daily Living) is a simple count variable and includes activities such as cooking, shopping, and driving. A higher IADL scale indicates a higher level of physical impairment. Table 6 in Appendix provides an overview of all variables.

## Results

**Table 1:** Rate of positive attitude by gender

	Men		Women		All		Gender difference test	
	Rate	*N*	Rate	*N*	Rate	*N*	$\chi ^2/{F}^{(\mathrm{a})}$	*p* value
Tablet	0.55	1113	0.47	1506	0.51	2619	19.61/11.81	0.00
Smartphone	0.63	1159	0.53	1588	0.58	2747	23.14/14.77	0.00
Social networks	0.31	1129	0.27	1552	0.29	2681	3.62/1.90	0.17
Voice-controlled PC	0.34	1073	0.26	1464	0.30	2537	21.26/11.55	0.00
Tracking system	0.59	1093	0.61	1518	0.60	2611	0.62/0.39	0.53
Auto fall alert	0.58	1091	0.65	1568	0.62	2659	13.22/8.20	0.00
Personal alarm	0.66	1109	0.72	1600	0.69	2709	10.25/6.50	0.01
Auto cooker control	0.45	1047	0.58	1501	0.52	2548	42.35/25.84	0.00

^a^Test statistics: uncorrected Chi-squared and design-based F

First descriptive statistics indicate that Personal Alarm systems enjoy the highest sympathy by respondents over 50 years (69% positive score) and are followed by Auto Fall Alerts with 62% positive attitudes. Tracking Systems and Smartphones also enjoy significantly positive rankings with 60 and 58% positive attitudes, respectively. Auto Cooker Control and Tablets are slightly in the middle with 52 and 51% shares of positive attitudes, respectively, while Social Networks and Voice-Controlled PCs exhibit the least positive attitudes with 30 and 29%, respectively. Table 1 lists the results by gender. The results indicate a more positive attitude towards entertainment applications by men, and a more positive attitude towards health applications by women. All gender differences are statistically significant at a 99% level according to chi-squared tests, except for Social Networks and Tracking Systems, which do not show significant gender differences.

**Fig. 1:**
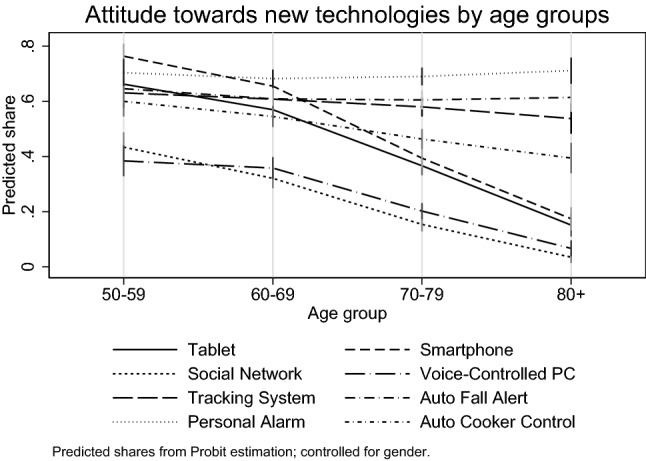
Share of positive attitudes by age groups

Based on Fig. 1, a very interesting age pattern exists. The results are derived from simple probit regressions, where we only control for age and gender to predict aggregate age patterns. We observe falling positive attitudes for *communication and entertainment* devices. However, a falling pattern for devices that are more *support and health* oriented is statistically insignificant. Specifically, a decrease with age is absent for the use of Auto Fall Alert and Personal Alarm. Conversely, attitudes towards Tablets or Smartphones decline significantly.

The results indicate that 77% of all respondents exhibit a positive attitude towards at least one new technology in the *health and support* group. Approximately, 69% display positive attitudes towards *communication and entertainment* technologies. Shares by gender show that 80% of females and 73% of males have a positive attitude towards *health and support* technology. With respect to *communication and entertainment* technologies, 73% of males and 66% of females report a positive attitude. The described gender differences are highly statistically significant.^4^ Approximately, 57% of all respondents are interested in both groups of technological devices, and 12% of the overall sample is not interested at all in new technologies.

**Fig. 2:**
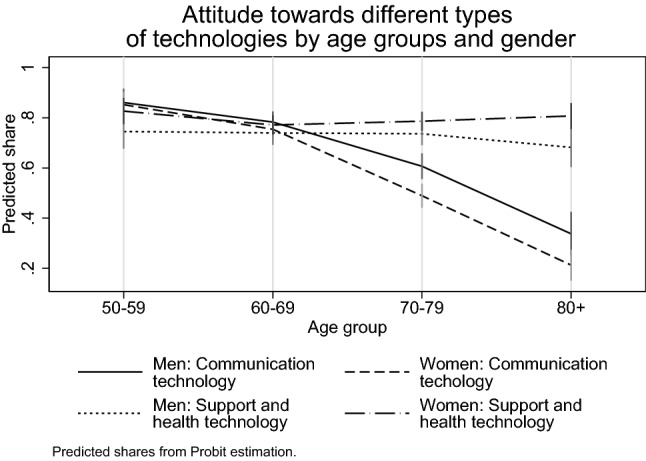
Share of positive attitudes by age groups and gender

The simple probit prediction model from Fig. 2 likewise reveals highly significant gender differences for both groups of technological devices. Women are more interested in *support and health* devices, while men are generally more interested in *communication and entertainment* devices. In both cases, the gender difference amounts to slightly over 5 percentage points at age means. We also observe a strong age effect for *communication and entertainment* devices, while barely any age effect is observed for *support and health* devices. This may indicate a growing demand for *support and health* devices with increasing age (and therefore also deteriorating health) that eventually compensates, or even overcompensates for the reasons for the age effect initially.

### Multivariate analysis

**Table 2:** Estimation results: Marginal effects from probit estimation with positive attitude towards different technologies as dichotomous depentent variable

	Communication & entertainment		Support & health	
	Men	Women	Men	Women
Age group (ref.: 50–59)				
60–69	$-$ 0.06	$-$ 0.05	0.06	$-$ 0.07
	(0.04)	(0.05)	(0.06)	(0.04)
70–79	$-$ 0.25***	$-$ 0.28***	0.05	$-$ 0.07
	(0.05)	(0.06)	(0.06)	(0.04)
80+	$-$ 0.47***	$-$ 0.48***	$-$ 0.01	$-$ 0.05
	(0.07)	(0.07)	(0.07)	(0.05)
Higher education (ref.: no)				
Yes	0.18***	0.18***	0.11**	0.06$+$
	(0.04)	(0.05)	(0.04)	(0.03)
Employment (ref.: retired/not employed)				
White collar	0.13*	0.18**	0.15**	$-$ 0.04
	(0.05)	(0.06)	(0.05)	(0.06)
Blue collar	0.01	0.02	0.17**	0.06
	(0.09)	(0.12)	(0.06)	(0.06)
Financial distress (ref.: no)				
Yes	$-$ 0.01	0.06	$-$ 0.06	$-$ 0.02
	(0.05)	(0.04)	(0.05)	(0.03)
Living in a house (ref.: no)				
Yes	0.01	0.04	0.08$+$	0.04
	(0.04)	(0.04)	(0.04)	(0.03)
Urban area (ref.: no)				
Yes	0.09*	0.03	0.02	0.02
	(0.04)	(0.04)	(0.04)	(0.03)
Partner in household (ref.: no)				
Yes	0.01	$-$ 0.02	0.04	$-$ 0.02
	(0.04)	(0.03)	(0.04)	(0.03)
Has children (ref.: no)				
Yes	0.07	0.02	0.04	0.01
	(0.05)	(0.05)	(0.05)	(0.04)
Poor or fair health (ref.: no)				
Yes	$-$ 0.05	$-$ 0.03	0.17***	0.06*
	(0.04)	(0.03)	(0.04)	(0.03)
# IADL limitations	$-$ 0.01	$-$ 0.06***	0.01	0.00
	(0.01)	(0.02)	(0.01)	(0.01)
Observations	1034	1372	1050	1461

Standard errors in parentheses. Marginal effects at means from probit estimation. +$(p<0.10)$, *$(p<0.05)$, **$(p<0.01)$, ***$(p<0.001)$

We perform a multivariate analysis in Table 2 to regress positive attitudes on our age dummies, education, basic employment indicators, and a few demographic factors. All variables included in the model are listed in the table. We also include two health indicators. The dependent variable is dichotomous, and thus, we use probit models separately for men and women. All analyses were performed using statistical software Stata/SE 13.0. We analyse positive or negative attitudes towards new technologies (all of which are dichotomous outcome variables) by using a probit estimation and report the marginal effects at means and corresponding standard errors. Significance levels are indicated by asterisks [$+(p<0.10)$, *$(p<0.05)$, **$(p<0.01)$, ***$(p<0.001)$].

The results confirm the above outlined age differences with respect to *communication and entertainment* devices. Specifically, individuals aged 70–79 years old are between 25 and 28 percentage points (male/female at means) and individuals aged 80+ years are from 47 to 48 percentage points less likely to value *communication and entertainment* devices compared to the youngest cohort. Education increases the odds of positive attitudes to new technologies by 11 to 18 percentage points, and this also holds for white-collar employment ($+13/+18$ percentage points). A single exception applies for women with respect to *support and health* devices in which neither education, nor white-collar employment exhibit any statistically significant effect. The result potentially points towards the overall importance (and need) of these types of devices for women irrespective of the educational level or the technological abilities acquired during employment.

Other factors that are statistically significant correspond to men living in urban areas as follows: the group of respondents is by 9 percentage points more inclined to value *communication and entertainment* devices positively. Fair or poor subjective health increases the positive stances towards support and health devices by 17 (male) and 6 (female) percentage points, respectively. Finally, physical limitation (IADL) decreases the positive stances towards *communication and entertainment* devices for women by 6 percentage points as always while holding all other variables constant at their mean values.

With respect to the level of individual devices or applications as indicated in Tables 4 and 5 in Appendix, our main results for age differences are completely identical, and the findings indicate that the largest negative differences arise for the use of Smartphones, followed by Tablets and Social Networks. Education positively affects almost all devices. Concerning white-collar workers, effects for females are smaller and more fragile. Men living in a household suffering from at least some financial distress tend to value health and support systems less. The financial situation of the household indicated by type of job and the financial distress variable appears to be more influential on males’ attitudes towards new technologies than females’, but those effects remain statistically insignificant. Men in urban areas are also more open-minded towards *communication and entertainment* devices.

Regarding health, particularly men are prone to technological devices for *health and support* use; if they display poor or fair health, their attitude increases significantly. Effects are only half as large for women. We performed all estimations with and without health variables. Results are not reported since the inclusion of health indicators did not considerably change the size and significance levels of coefficients of other variables. This finding also applies to the financial distress variable, that does not bear any effect on the general outcome.

## Discussion and conclusion

The analyses in the present study indicate a positive attitude of a majority of respondents in Austria towards most technological innovations with the potential to cover specific needs of an ageing population. This is specifically true for devices and applications in the *support and health* group, such as Personal Alarms, Auto Fall Alerts, and Tracking Systems. A more heterogeneous picture emerges for devices and applications in the *communication and entertainment* group. With the exception of Smartphones and Tablet computers, the overall attitude towards innovations such as Voice-Controlled Computers or Social Networks is rather negative. The concrete purposes and functions of devices and applications appear to drive respondents’ attitudes as follows: the more precise the functions, the higher the acceptance rate, in general.

Specifically, a significant gender gap emerges between the two groups of applications and devices defined in the study as follows: women appreciate devices in the *support and health* group more. Conversely, men value *communication and entertainment* innovations more. This finding appears to indicate a general pattern in the appreciation for new technologies: Our results show that women value technologies with considerably concrete purposes and functions more when compared with men. However, the extent of the gender gap is limited, and in most cases does not change the overall picture where *support and health* technologies are generally more appreciated than their counterparts. Our research design allows us to differentiate between *communication* and *support and health* items; the previous literature (e.g. Gell et al. 2013) finds that men value *communication technologies* more. We can show that gender differences depend on the type of technology: for communication, men are more appreciative, whereas in the case of *support and health technologies*, women are ahead.

Our analysis also indicates a pronounced age effect for *communication and entertainment* devices. However, at the present stage—using only a cross section of data—we are unable to distinguish whether we are confronted with genuine age effects, in which older individuals are generally less interested in *communication and entertainment* devices, or if it is more a matter of cohort effects. A plausible explication for less interest in technological devices at older ages might also be rooted in less acquaintance and experience of those generations (during their lifetime) with technological devices in general, and *communication and entertainment* devices in particular. If so, the presumed cohort effects are expected to fade in the forthcoming years.

Most importantly, age or cohort effects are not applicable to *support and health* devices. An alternative argument is that with increase in age, possible age effects are offset by a higher need of (technological) assistance due to deteriorating health and other conditions. However, our results also hold if we control for the state of health and limitations in instrumental activities of daily living. The control variables are assumed to capture any effect originating from deteriorating health and other limitations that increase with old age although they do not significantly alter the age effect. We are therefore inclined to conclude that in contrast to *communication and entertainment* devices, *support and health* devices generally do not exhibit age effects. This is a new finding in the literature. Previous research (e.g. Gell et al. 2013; Vorrink et al. 2017 or Keränen et al. 2017) gave the impression that the use of new technology decreases with age. We can show that the use of communication technology, indeed, decreases with age, but the use of support or health technologies does not decrease at all.

*Limitations* of the study include the fact that we cannot distinguish age from cohort effects, generalisability across nations and additional use of socio-economic characteristics. With respect to age effects in cross-sectional data, it is not possible to accurately distinguish between age and cohort effects. The use of a device must be learned and trained, and thus it appears highly unlikely that the attitude towards such devices should decrease with rising age. It is potentially more likely that older cohorts of respondents are generally less technology-prone than younger cohorts, latter being generally much more in contact with modern technology during their lifetime. Such an interpretation also leads to different predictions with respect to ageing individuals, and we forecast similarly high positive attitudes for individuals in the age group of 80+ years in 30 years as we currently measure for individuals in the age group of 50–59 years. Specifically, the forecast potentially increases given additional exposure and better usage possibilities.

We conclude that in particular, technological innovations that are categorized as *supportive tools*, or with *specific health purposes*, are the ones with the highest potential of being positively appreciated and received by elderly members of society.

## Appendix

See Tables 3, 4, 5, 6.

**Table 3:** Attitudes towards new technologies: Detailed selection

	I don’t know this	I am already using this	I am open to this	This is/would be great help	I find this daunting	I doubt that I would find this helpful	I am not interested in this	I do not feel comfortable around this
Tablet	0.12	0.20	0.24	0.03	0.01	0.04	0.34	0.09
Smartphone	0.08	0.35	0.18	0.02	0.01	0.04	0.31	0.08
Social networks	0.09	0.19	0.08	0.01	0.03	0.06	0.54	0.05
Voice-controlled PC	0.16	0.04	0.19	0.02	0.01	0.07	0.49	0.05
Tracking system	0.13	0.03	0.44	0.07	0.01	0.05	0.26	0.02
Auto fall alert	0.10	0.01	0.48	0.09	0.01	0.04	0.28	0.02
Personal alarm	0.08	0.03	0.53	0.09	0.01	0.04	0.22	0.01
Auto cooker control	0.17	0.02	0.37	0.06	0.01	0.05	0.33	0.02
*N*	3085	3085	3085	3085	3085	3085	3085	3085

Weighted; Multiple answers possible; Concurrent positive and negative attitudes included

**Table 4:** Estimations with binary on positive attitude for each technological device for males

	Tablet	Smartphone	Social Networks	Voice-Controlled PC	Tracking System	Auto Fall Alert	Personal Alarm	Auto Cooker Control
Age group (ref.: 50–59)								
60–69	$-$ 0.10	$-$ 0.11*	$-$ 0.07	0.03	0.06	0.04	0.06	0.10
70–79	$-$ 0.27***	$-$ 0.31***	$-$ 0.23***	$-$ 0.11	0.04	$-$ 0.00	0.02	$-$ 0.00
80+	$-$ 0.40***	$-$ 0.51***	$-$ 0.35***	$-$ 0.27***	$-$ 0.05	$-$ 0.05	0.02	$-$ 0.08
Higher education (ref.: no)								
Yes	0.20***	0.21***	0.07	0.20***	0.15***	0.08$+$	0.07	0.03
Employment (ref.: not employed/retired)								
White collar	0.20**	0.20***	0.08	0.20**	0.19**	0.17*	0.15*	0.25***
Blue collar	$-$ 0.06	$-$ 0.07	0.01	0.06	0.08	0.13	0.17*	0.08
Financial distress (ref.: no)								
Yes	$-$ 0.05	$-$ 0.01	$-$ 0.02	$-$ 0.02	$-$ 0.11$+$	$-$ 0.16*	$-$ 0.10$+$	$-$ 0.10
Living in a house (ref.: no)								
Yes	0.07	0.01	$-$ 0.02	$-$ 0.06	0.03	0.02	0.03	$-$ 0.05
Urban area								
Yes	0.10*	0.10*	0.09$+$	0.00	$-$ 0.05	$-$ 0.02	0.01	$-$ 0.01
Partner in household (ref.: no)								
Yes	0.03	$-$ 0.02	$-$ 0.08	$-$ 0.01	0.10$+$	0.03	0.03	0.11*
Has children (ref.: no)								
Yes	0.04	0.01	0.08	0.05	0.04	0.04	0.03	0.06
Poor or fair health (ref.: no)								
Yes	$-$ 0.05	$-$ 0.06	$-$ 0.06	$-$ 0.00	0.11*	0.15***	0.16***	0.10$+$
# IADL limitations	$-$ 0.02	$-$ 0.04*	$-$ 0.04	$-$ 0.00	$-$ 0.00	0.03$+$	0.02	$-$ 0.02
Observations	1022	1065	1038	981	1007	1000	1017	955

Marginal effects at means from probit estimation. + $(p<0.10)$, *$(p<0.05)$, **$(p<0.01)$, ***$(p<0.001)$

**Table 5:** Estimations with binary on positive attitude for each technological device for females

	Tablet	Smartphone	Social networks	Voice-controlled PC	Tracking system	Auto fall alert	Personal alarm	Auto cooker control
Age group (ref.: 50-59)								
60–69	$-$ 0.03	$-$ 0.05	$-$ 0.13*	$-$ 0.01	$-$ 0.03	$-$ 0.06	$-$ 0.02	$-$ 0.16**
70–79	$-$ 0.25***	$-$ 0.33***	$-$ 0.29***	$-$ 0.16**	$-$ 0.07	$-$ 0.05	$-$ 0.01	$-$ 0.20***
80+	$-$ 0.42***	$-$ 0.47***	$-$ 0.36***	$-$ 0.24***	$-$ 0.05	$-$ 0.05	0.00	$-$ 0.22**
Higher education (ref.: no)								
Yes	0.20***	0.21***	0.10**	0.15***	0.08$+$	0.05	0.02	0.06
Employment (ref.: not employed/retired)								
White collar	0.09	0.25***	0.07	0.08	0.04	$-$ 0.01	0.02	0.01
Blue collar	0.01	0.02	$-$ 0.02	$-$ 0.10$+$	0.09	$-$ 0.02	0.06	0.04
Financial distress (ref.: no)								
Yes	0.04	0.06	0.06	0.01	$-$ 0.04	$-$ 0.07	$-$ 0.04	$-$ 0.04
Living in a house (ref.: no)								
Yes	0.05	0.05	$-$ 0.01	$-$ 0.05	0.04	0.02	0.04	0.01
Urban area								
Yes	0.04	0.05	0.06	$-$ 0.01	0.00	$-$ 0.03	$-$ 0.00	$-$ 0.02
Partner in household (ref.: no)								
Yes	0.05	$-$ 0.02	$-$ 0.02	$-$ 0.01	$-$ 0.05	$-$ 0.02	$-$ 0.04	0.08*
Has children (ref.: no)								
Yes	0.03	0.04	0.02	0.00	0.02	0.00	$-$ 0.01	0.03
Poor or fair health (ref.: no)								
Yes	0.01	$-$ 0.06	$-$ 0.00	$-$ 0.04	0.03	0.08*	0.08*	0.07$+$
# IADL limitations	$-$ 0.08***	$-$ 0.05**	$-$ 0.04$+$	$-$ 0.03	$-$ 0.01	$-$ 0.00	0.00	0.00
Observations	1364	1435	1402	1321	1369	1413	1443	1351

Marginal effects at means from probit estimation. +$(p<0.10)$, *$(p<0.05)$, **$(p<0.01)$, ***$(p<0.001)$

**Table 6:** Summary statistics, not weighted

Variable	Description	*N*	Median	Mean	SD	Min	Max
*Demographics*							
Year of birth		3085	1946	1946	9.353	1912	1964
Age	Age in 2015	3085	69	69.44	9.353	51	103
Gender	$1 =$ Female	3085		0.586		0	1
Age group 50–59		3085		0.160		0	1
Age group 60–69		3085		0.362		0	1
Age group 70–79		3085		0.330		0	1
Age group 80+		3085		0.147		0	1
*Family and living situation*							
Higher education	1 $=$ Obtained higher education entrance qualification	3020		0.237		0	1
Financial distress	1 $=$ Able to make ends meet with difficulty	3049		0.159		0	1
Partner in household	1 $=$ Partner is living in household	3085		0.643		0	1
Has children	1 $=$ has children	3084		0.887		0	1
Living in a house	1 $=$ Living in a house	2936		0.603		0	1
Urban area	1 $=$ Urban area	2925		0.550		0	1
*Employment*							
Retired	1 $=$ Retired	3048		0.742		0	1
Unemployed	1 $=$ Unemployed	3048		0.0138		0	1
Homemaker	1 $=$ Homemaker	3048		0.0876		0	1
Employed	1 $=$ Employed or selfemployed	3048		0.143		0	1
White collar	1 $=$ White-collar worker	3026		0.102		0	1
Blue collar	1 $=$ Blue-collar worker	3026		0.035		0	1
*Health*							
Poor or fair health	1 $=$ Poor or fair self-assessed health	3085		0.338		0	1
# IADL limitations	Number of limitations in instrumented activities of daily living	3084	0	0.616	1.640	0	9
*Technology*							
Tablet^a^	1 $=$ Positive attitude	2619		0.460		0	1
Smartphone^a^	1 $=$ Positive attitude	2747		0.514		0	1
Social networks^a^	1 $=$ Positive attitude	2681		0.249		0	1
Voice-controlled PC^a^	1 $=$ Positive attitude	2537		0.260		0	1
Tracking system^a^	1 $=$ Positive attitude	2611		0.581		0	1
Auto fall alert^a^	1 $=$ Positive attitude	2659		0.601		0	1
Personal alarm^a^	1 $=$ Positive attitude	2709		0.682		0	1
Auto cooker control^a^	1 $=$ Positive attitude	2548		0.502		0	1
Communication and entertainment technology	1 $=$ Positive attitude towards Tablet, Smartphone, Social Networks or Voice-Controlled PC	2639		0.642		0	1
Support and health technology	1 $=$ Positive attitude towards Tracking System, Auto Fall Alert, Personal Alarm or Auto Cooker Control	2766		0.758		0	1
*Weight*							
Weight	Calibrated cross-sectional individual weight—wave 6	3085	786.7	968.2	764.5	74.09	8284

All obsverations 50+ of wave 6 that returned the drop off questionnaire and where weights are available

^a^For detailed and not cleaned answers see Table 3

## Footnotes

1 The paper questionnaire can be accessed here: www.share-project.org/fileadmin/pdf_questionnaire_wave_6/AT_EN_drop-off_w6.pdf.

2 An Emergency Tracking Systems is a wristband with an emergency button, that connects individuals directly to a 24-hour call centre.

3 The number of excluded cases ranges between 10 and 52 for each technological device with an average of 28. Auto Cooker Control exhibits the lowest number of invalid answers, and Tablets correspond to the maximum number of invalid answers. In the first column of Table 3, details on ‘do not know’ answers are included.

4 Pearsons chi-squared test statistics of gender differences: *Communication and entertainment* technologies (chi-squared = 16.51, design-based $F = 11.69$, *p* value = 0.00) and *health and support* technologies (chi-squared = 14.92, design-based $F = 9.96$, *p* value = 0.00)
